# The capacity of short-chain fructo-oligosaccharides to stimulate faecal bifidobacteria: a dose-response relationship study in healthy humans

**DOI:** 10.1186/1475-2891-5-8

**Published:** 2006-03-28

**Authors:** Yoram Bouhnik, Laurent Raskine, Guy Simoneau, Damien Paineau, Francis Bornet

**Affiliations:** 1 Hépato-Gastroentérologie et Assistance Nutritive, Hôpital Lariboisière, 2 rue Ambroise Paré, 75475 Paris Cedex 10, France; 2 Bactériologie – Virologie, Hôpital Lariboisière, 2 rue Ambroise Paré, 75475 Paris Cedex 10, France; 3 Unité de Recherche Thérapeutique, Hôpital Lariboisière, 2 rue Ambroise Paré, 75475 Paris Cedex 10, France; 4 Nutri-Health SA, 3 avenue Paul Doumer, 92500 Rueil-Malmaison, France

## Abstract

**Background:**

Short-chain fructo-oligosaccharides (scFOS) are well-known for their bifidogenicity. In a large study comprising 200 healthy volunteers, we determined the bifidogenic properties of 7 non-digestible carbohydrates administered at a dose of 10 g/d in the diet; we analysed dose-response relationships of the bifidogenic substrates at doses ranging from 2.5 to 10 g/d in comparison with a placebo. The aim of this presentation is to give more details about the dose-response effects of short-chain fructo-oligosaccharides (scFOS).

**Methods:**

Forty healthy volunteers (18 males, 22 females) eating their usual diets were randomly divided into 5 groups of 8 subjects and received scFOS at a dose of 2.5, 5.0, 7.5 and 10 g/d or a placebo for 7 d. Stools were collected before (day (d) 8) and at the end (day (d) 15) of sugar consumption, and tolerance was evaluated using a daily chart.

**Results (m ± SEM):**

Bifidobacteria counts increase was higher in scFOS than in placebo group for all doses tested [2.5 g/d (from 9.15 ± 0.59 to 9.39 ± 0.70; P = 0.02); 5 g/d (from 10.21 ± 0.21 to 10.67 ± 0.22; P = 0.03); 7.5 g/d (from 9.28 ± 0.49 to 9.85 ± 0.35;P = 0.01); 10 g/d (from 9.00 ± 0.81 to 10.18 ± 0.60; P = 0.003)]. A significant correlation between the ingested dose of scFOS and faecal bifidobacteria counts was observed at d15 (r^2 ^= 0.307, P < 0.001). Total anaerobes increased at the dose of 10 g/d. No significant differences were found for Bacteroides, Lactobacillus, enterobacteria or pH in any group. The frequency of digestive symptoms was not different between scFOS at any of the doses tested and placebo. Bloating was significantly more intense during scFOS ingestion at doses of 2.5 and 5 g/d, but not at doses of 7.5 and 10 g/d. Excess flatus, borborygmi and abdominal pain did not differ from the placebo at any of the doses tested.

**Conclusion:**

This study showed that scFOS is bifidogenic and well tolerated at doses ranging from 2.5 to 10 g/d, and that there is a dose-response relationship in healthy volunteers.

## Background

Short chain fructo-oligosaccharides (scFOS) are a mixture of oligosaccharides consisting of glucose linked to fructose units; links between fructose units are β-(1,2) [1]. They are produced commercially from sucrose using an enzymatic process. ScFOS are poorly digested in the human small intestine but are fermented in the colon by the resident microflora [2].

In light of the recent interest in "prebiotics", defined as "a non-digestible food ingredient that beneficially affects the host by selectively stimulating the growth and/or the activity of one or a limited number of bacterial species in the colon" [3], it has been shown in humans that addition to the diet of short-chain fructo-oligosaccharides (scFOS) at doses of from 4 to 12.5 g/d leads to an increase in faecal bifidobacteria counts [4,5]. Such bifidobacteria-promoting dietary interventions can be perceived as beneficial, since bifidobacteria are a saccharolytic genus and could contribute to the same type of protection that breast-feeding provides against gut infections [6], while also playing a role in the prevention of colorectal carcinogenesis [4,5].

In a recent study comprising 200 healthy volunteers, we determined the bifidogenic properties of 7 non-digestible carbohydrates administered at a dose of 10 g/d in the human diet : Four non-digestible carbohydrates were found to be statistically different from placebo, i.e. bifidogenic, at 10 g/d: scFOS, soybean-oligosaccharides, galacto-oligosaccharides and type III resistant starch; we therefore performed a dose-response relationship analysis of these 4 substrates at doses ranging from 2.5 to 10 g/d and compared them to a placebo [7]. The effects of the 7-day treatment period were found to be significantly different among the 4 non-digestible carbohydrates (P = 0.009). A trend was found for the doses tested (P= 0.06) suggesting that the relationship between dose and bifidobacteria count could be different among the 4 non-digestible carbohydrates, and that among them, a dose-response effect could be present. Since we previously demonstrated a dose-response relationship between scFOS and bifidobacteria at higher doses [5], we considered it of interest to investigate a possible dose-response relationship between scFOS and bifidobacteria at lower doses.

## Methods

### Subjects

Forty healthy volunteers [18 males, 22 females, age 29 ± 1.3 (mean ± SEM)] were included. Exclusion criteria were: history of gastrointestinal disease except for appendectomy; antibiotics or laxatives taken during the two months before the study; use of other medication during the investigation period. Subjects gave written informed consent to take part in the study, which was performed in accordance with local legislation, the ICH guidelines and the principles laid down in the current version of the Declaration of Helsinki, and was approved by an Ethics Committee (the "Comité Consultatif pour la Protection des Personnes se prêtant à la Recherche Biomédicale" of Saint Louis Hospital, Paris, France).

The scFOS tested was Actilight™ [Beghin Meiji, Paris, France, comprising 44% 1-kestose (GF2), 46% nystose (GF3) and 10% 1F-β-fructofuranosyl nystose (GF4)]; the placebo was 50% sucrose – 50% fully digestible waxy maize-derived maltodextrins (DE6.5) (Cerestar, Vilvoorde, Belgium).

### Experimental design

To evaluate possible dose-response effects on the intestinal microflora, 40 volunteers were randomized to 5 groups of 8 subjects and they ingested scFOS at a daily dose of 2.5, 5.0, 7.5 or 10 g/d from d8 to d14 or the placebo in two oral doses after lunch and dinner. All subjects consumed their usual daily diet from the pre-inclusion day (d0) to the end of the study (d15). They were instructed to exclude from their diet fermented dairy products containing viable bifidobacteria and to limit consumption of foods containing high levels of non-digestible oligosaccharides such as onion, asparagus, wheat, rye, triticale and Jerusalem artichoke.

### Digestive symptoms

Gastrointestinal side effects were evaluated using a daily chart in which symptoms (excess flatus, borborygmi, bloating, abdominal pain) were graded from 0 (no symptoms) to 3 (severe symptoms). The frequency and consistency of stools were also noted by the volunteers and diarrhoea was defined as one or more watery stools, or more than 3 stools per day.

### Stool collection

Stools were recovered twice, on the morning of d8 before the start of scFOS consumption, and on the morning of d15, i.e. after 7d of scFOS consumption (d8 to d14). Samples were collected in plastic containers rendered anaerobic (Anaerocult A; Merck, Darmstadt, Germany), immediately stored at 4°C, transferred to the laboratory and analyzed. The procedure from stool emission to bacteriological analysis lasted less than 1 hour.

### Bacterial counts and pH

Faecal samples (1 g) were introduced in the first pre-weighed tube of the dilution series and thoroughly mixed and then further ten-fold dilutions were made in a reduced diluent (cysteinated 1/4 strength Ringer diluent). 0.1 ml of each dilution was plated in different selective media to outnumber several bacterial genera: total anaerobic counts (Wilkins-Chalgren agar), Bifidobacterium (Beerens' medium), Lactobacillus (MRS agar), Bacteroides (BBE agar) and Enterobacteria (McConkey agar). The tests were duplicated for the first two media. Plates of the first three media were incubated anaerobically for 5 to 7 days, MRS agar for 48 hours under atmosphere enriched in CO2 and McConkey agar aerobically for 48 hours. Colony counts were obtained and expressed as a log of the colony-forming units (CFU) per gram of fresh faeces. At the same time, the fresh stool pH was measured by pH meter (Bioblock, Illkirch, France).

### Data analysis

The descriptive statistics used the mean and standard error of the mean (m ± sem).

Efficacy analysis: The comparisons between each dose of scFOS versus placebo were performed on variations of bacteria counts (differences after-before treatment for bifidobacteria and other bacteria) using the unpaired t-test. Since the difference between days 8 to 15 compared with placebo was the reference, p-values are not reported.

A dose-response relationship was therefore sought using the linear regression model.

Tolerance analysis: a global analysis of the observed frequencies was performed for the 5 groups during the treatment period and for each symptom using a chi^2 ^test.

Symptom intensity was recorded every day as follows: 0: no symptoms; 1: mild symptoms; 2: moderate symptoms; 3: severe symptoms, resulting in a daily score.

All daily scores were added for each symptom 1) before the treatment period from d1 to d8 (a.m.) and 2) during the treatment period from d8 (p.m.) to d15; in order to test the differences (doses versus placebo) on variation in total score (differences During-Before treatment). The same procedure was used (ANOVA, Fisher's test).

## Results

### Faecal bacterial counts and pH

Bifidobacteria counts increased in the scFOS groups at the doses of 2.5, 5, 7.5 and 10 g/d. Total anaerobes increased at the dose of 10 g/d (Table 1). No significant differences were found for Bacteroides, Lactobacillus, enterobacteria and pH in any group (Table 2).

**Table 1:** Faecal bifidobacteria and total anaerobe counts (m ± SEM, log cfu/g) in 40 healthy volunteers assigned to a 7-d consumption of short-chain fructo-oligosaccharides (scFOS) at a dose from 2.5 to 10 g/d or a placebo

scFOS dose (g/d)	Total anaerobes				Bifidobacteria			
	
	d8*	d15*	Δd15-d8	P**	d8*	d15*	Δ d15-d8	P**
0 (placebo)	12.59 ± 0.17	12.54 ± 0.12	- 0.05 ± 0.16	***	10.06 ± 0.29	9.57 ± 0.21	- 0.49 ± 0.23	***
2.5	12.32 ± 0.21	12.55 ± 0.14	+ 0.22 ± 0.19	0.29	9.15 ± 0.59	9.39 ± 0.70	+ 0.24 ± 0.16	0.02
5.0	12.48 ± 0.18	12.66 ± 0.14	+ 0.17 ± 0.28	0.51	10.21 ± 0.21	10.67 ± 0.22	+ 0.46 ± 0.31	0.03
7.5	12.45 ± 0.16	12.55 ± 0.13	+ 0.09 ± 0.06	0.42	9.28 ± 0.49	9.85 ± 0.35	+ 0.57 ± 0.25	0.01
10	11.65 ± 0.37	12.68 ± 0.14	+ 1.04 ± 0.41	0.03	9.00 ± 0.81	10.18 ± 0.60	+ 1.18 ± 0.42	0.003

*days 1–8 was a run-in period during which no treatment occurred, but subjects excluded from their diet fermented dairy products containing viable bifidobacteria and limited consumption of food products containing high level of non-digestible oligosaccharides.

** Statistical analyses were performed versus placebo, on the difference d15-d8, using an unpaired t-test

*** As the difference days 15-8 with placebo was the reference, p-values are not reported.

**Table 2:** Bacterial counts and pH (m ± SEM, log cfu/g) in 40 healthy volunteers assigned to a 7-d consumption of short-chain fructo-oligosaccharides (scFOS) at a dose from 2.5 to 10 g/d or a placebo

scFOS dose (g/d)	Lactobacillus			Bacteroides			Enterobacteria			pH		
	
	d8*	d15*	P**	d8*	d15*	P**	d8*	d15*	P**	d8*	d15*	P**
0 (placebo)	5.21 ± 0.36	5.18 ± 0.41	***	8.76 ± 0.45	8.53 ± 0.32	***	6.93 ± 0.29	7.07 ± 0.37	***	6.94 ± 0.13	6.91 ± 0.11	***
2.5	4.66 ± 0.48	5.14 ± 0.60	NS	8.93 ± 0.28	9.08 ± 0.17	NS	6.93 ± 0.26	7.28 ± 0.33	NS	6.68 ± 0.18	6.75 ± 0.14	NS
5.0	5.34 ± 0.66	5.99 ± 0.67	NS	8.87 ± 0.18	9.09 ± 0.28	NS	7.34 ± 0.33	7.81 ± 0.32	NS	6.48 ± 0.15	6.52 ± 0.11	NS
7.5	5.37 ± 0.40	5.97 ± 0.39	NS	8.74 ± 0.25	8.61 ± 0.29	NS	6.30 ± 0.29	6. 78 ± 0.26	NS	6.66 ± 0.13	6. 78 ± 0.13	NS
10	5.40 ± 0.41	5.73 ± 0.83	NS	8.51 ± 0.41	9. 39 ± 0.26	NS	6.28 ± 0.40	6.58 ± 0.32	NS	7.05 ± 0.21	7.20 ± 0.25	NS

*days 1–8 was a run-in period during which no treatment occurred, but subjects excluded from their diet fermented dairy products containing viable bifidobacteria and limited consumption of food products containing high level of non-digestible oligosaccharides.

** Statistical analyses were performed versus placebo, on the difference d15-d8, using an unpaired t-test

*** As the difference days 15-8 with placebo was the reference, p-values are not reported.

### Dose-effect of scFOS on bifidobacteria concentrations

**Figure 1:**
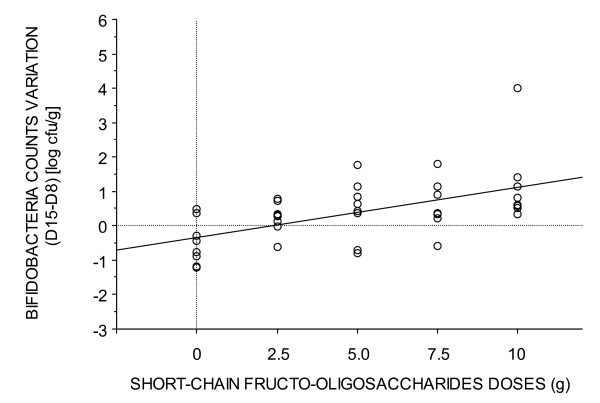
Correlation between the dose of ingested scFOS and the faecal bifidobacteria counts on the d15 – d8 difference.

Bifidobacteria counts did not differ significantly among the groups at d1. A significant correlation between the dose of ingested scFOS and faecal bifidobacteria counts was observed on the d15 – d8 difference (r2= 0.307, P < 0.001) (Figure 1).

### Digestive tolerance

Digestive symptom frequency, as assessed by cumulative daily scores, did not differ between placebo and scFOS at all doses tested (Table 3). Bloating was significantly more intense during scFOS ingestion at doses of 2.5 and 5 g/d, but not at doses of 7.5 and 10 g/d (Table 4). Excess flatus, borborygmi and abdominal pains were no different from placebo at all doses tested (Table 4). No diarrhoea was reported by any subjects.

**Table 3:** Observed frequencies for digestive symptoms (n and % of column totals), during the treatment period in 40 healthy volunteers assigned to a 7-d consumption of short-chain fructo-oligosaccharides (scFOS) at a dose from 2.5 to 10 g/d or a placebo

scFOS dose (g/d)	Excess flatus		Bloating		Borborygmi		Abdominal pain	
	
	n	%	n	%	n	%	n	%
0 (placebo)	6/8	75	5/8	62.5	4/8	50	3/8	37.5
2.5	7/8	87.5	6/8	75	5/8	62.5	4/8	50
5.0	6/8	75	4/8	50	5/8	62.5	3/8	37.5
7.5	7/8	87.5	4/8	50	3/8	37.5	5/8	62.5
10	7/8	87.5	6/8	75	4/8	50	5/8	62.5

**Table 4:** Intensity of digestive symptoms (scores) in 40 healthy volunteers assigned to a 7-d consumption of short-chain fructo-oligosaccharides (scFOS) at a dose from 2.5 to 10 g/d or a placebo

scFOS dose (g/d)	Excess flatus			Bloating			Borborygmi			Abdominal pain		
	
	d1-d8*	d8-d15*	P**	d1-d8*	d8-d15*	P**	d1-d8*	d8-d15*	P**	d1-d8*	d8-d15*	P**
0 (placebo)	4.12 ± 1.35	4.12 ± 1.45	***	3.87 ± 1.80	2.62 ± 0.92	***	3.25 ± 1.80	2.75 ± 1.47	***	1.50 ± 1.00	0.50 ± 0.26	***
2.5	1.75 ± 0.45	3.62 ± 1.28	NS	1.50 ± 0.42	3.12 ± 1.28	0.03	1.37 ± 0.49	300 ± 1.36	NS	1.12 ± 0.61	1.12 ± 0.61	NS
5.0	3.00 ± 1.08	5.25 ± 1.71	NS	1.75 ± 0.97	3.37 ± 1.76	0.03	1.75 ± 0.92	2.87 ± 1.42	NS	0.87 ± 0.51	0.87 ± 0.51	NS
7.5	3.50 ± 0.86	3.50 ± 0.68	NS	1.75 ± 1.03	1.50 ± 0.62	NS	1.75 ± 0.64	0.62 ± 0.32	NS	2.12 ± 0.87	2.12 ± 0.87	NS
10	3.37 ± 1.16	4.75 ± 1.03	NS	2.25 ± 1.06	2.62 ± 1.16	NS	1.50 ± 0.75	1.62 ± 0.86	NS	2.12 ± 0.81	2.12 ± 0.81	NS

*days 1–8 was a run-in period during which no treatment occurred, but subjects excluded from their diet fermented dairy products containing viable bifidobacteria and limited consumption of food products containing high level of non-digestible oligosaccharides.

** Statistical analyses were performed versus placebo, on the difference d15-d8, using Fisher's Test

*** As the difference days 15-8 with placebo was the reference, p-values are not reported. Symptom intensity was noted every day using a 4-grade scale 0: no symptom; 1: mild symptoms; 2: moderate symptoms; 3: severe symptoms. Cumulative daily scores were calculated for each period [d1, d8 a.m.], [d8 p.m., d15] and are reported in the present table as m ± sem. Severity of symptoms and cumulative daily scores can be related via the following scale: 0: no symptom; 1–7: mild symptoms; 8–14: moderate symptoms; 15–21: severe symptoms.

## Discussion

This placebo-controlled dose-response study shows that 7 days of ingestion of scFOS at a dose of 2.5 to 10 g/d, which was well tolerated, led to a significant increase in faecal bifidobacteria in healthy volunteers. This is the first study to demonstrate the bifidogenic effect of sc-FOS with such a low dose (2.5 g/d). Moreover, it must be stressed that the increase in bifidobacteria counts was correlated with the dose of ingested scFOS. We previously reported such an effect in healthy volunteers in another dose-response relationship study. However, the optimal and well-tolerated dose of scFOS leading to a significant increase in faecal bifidobacteria under usual diet was 10 g/d. These differences are probably due to the relatively small size of each sample, which decreased the power of the study. The increase in total anaerobes observed at the dose of 10 g/d is probably due to the high stimulation of colonic microflora induced by prebiotics [8,9]. The faecal bifidobacteria and anaerobe levels observed in this study were similar to those found in previous studies [3,5] in healthy volunteers on scFOS.

ScFOS has been studied extensively and its bifidogenic effects demonstrated in well-controlled human trials [3-5,8]. However, Roberfroid et al. concluded from a compilation work that in a large population, there seems to be no dose-response effect of these non-digestible carbohydrates on bifidobacteria for daily intake doses between 4 and 40 g/d [10]. Here, we found a linear dose-response relationship from 2.5 to 10 g/d, suggesting a dose-effect relationship. Such a relationship has been previously found in a study with scFOS, in which the range of doses was wider, from 5 to 20 g/d [8], and in another trial using a mixture of galacto- and fructooligosaccharides as supplementation of term infant formula [11]. It was not found with other substrates such as galacto-oligosaccharides alone, resistant starch or soybean oligosaccharides [7].

We did not find any significant reduction in any other genus. There is little published data in humans available in the literature for comparison. Contradictory results have been reported in this field; while Gibson et al. [3] showed a significant reduction in Bacteroides using 15 g/d oligofructose, Rao [9] found an increase in Bacteroides using oligofructose at dose of 5 g/d. Moreover using oligofructose at 8 g/d, 2 authors did not find any effect on Bacteroide [7,12]. The reasons for these discrepancies are unclear.

A decrease in colonic pH could reduce the risk of developing colonic cancer, since an inverse correlation between stool pH and colon cancer risk was observed [13,14]. Slight acidification of faecal contents during scFOS ingestion was observed in animals [15] and in humans [12]. In our previous studies, faecal pH did not change during ingestion of scFOS [5,8]. However, as the faecal pH is the net sum of the degree of short-chain fatty acid absorption and bicarbonate secretion during passage through the colon, faecal pH does not reflect the pH in the colon under physiological conditions [16,17].

Symptoms relating to gas production in the gut are widely reported in human prebiotic feeding studies but nevertheless remain very mild at the recommended intake levels [18,19]. Compared to placebo, we did not find any significant digestive intolerance symptoms except minor bloating with scFOS. Similarly, no dose-response relationship for digestive symptoms was observed. In a threshold study to evaluate symptomatic response to varying levels of scFOS ingested regularly by 14 healthy volunteers, excessive flatus and borborygmi were reported by about 10% of volunteers at 10 g/d of scFOS, and excessive flatus, borborygmi and bloating were recorded for about 20–30% of volunteers at 20 g/d [20]. In another study in which 10 volunteers ingested 15 g/d FOS for 12 days, gaseous symptoms such as abdominal cramps, excess flatus and bloating were all significantly more severe in subjects ingesting the FOS than in control subjects ingesting sucrose (P < 0.05) [21]. However, with the exception of flatulence, these symptoms, if present, were usually mild, and did not increase (or decrease) during the course of the 12-day period. In our previous study comprising 10 healthy volunteers who ingested 12.5 g/d of scFOS for 12 days, only bloating was found to be significantly more frequent during the scFOS ingestion period than during placebo ingestion (P < 0.05), but this was very mild and present in only 5/10 volunteers [5]. From all these results, it appears that the most common symptoms noted during scFOS administration are excess flatus and/or bloating, but only a minority of subjects experiences them and they are usually very mild.

## Conclusion

This study confirmed that scFOS is bifidogenic and well tolerated in healthy volunteers. A bifidogenic effect appeared for the first time at 2.5 g/d of scFOS, and a dose-response relationship was demonstrated from 2.5 to 10 g/d.

## Competing interests

Yoram Bouhnik, Laurent Raskine, Guy Simoneau: no affiliations Damien Paineau, Francis Bornet: Nutri-Health SA, Rueil-Malmaison, France

## Authors' contributions

YB participated in the study design, the data collection, the data analysis and he drafted the manuscript. LR carried out the data collection. GS participated in the data collection and analysis. DP participated in the writing of the manuscript. FB conceived of the study and participated in the writing of the manuscript. All authors read and approved the final manuscript.
